# The acupuncture-related therapy for stable chronic obstructive pulmonary disease: A protocol for systematic review and network meta-analysis

**DOI:** 10.1097/MD.0000000000028832

**Published:** 2022-02-11

**Authors:** Zengtu Li, Jiali Lou, Chao Wang, Haijuan Zhang

**Affiliations:** a Department of Tuina, The Third Affiliated Hospital of Zhejiang Chinese Medical University, Hangzhou City, Zhejiang Province, China; b Department of Acupuncture and Moxibustion, First People's Hospital of Xiaoshan District, Hangzhou City, China; c Department of Acupuncture and Moxibustion, The Third Affiliated Hospital of Zhejiang Chinese Medical University, Hangzhou City, China; d The Third Clinical Medical College, Key Laboratory of Acupuncture and Neurology of Zhejiang Province, Zhejiang Chinese Medical University, Hangzhou City, China.

**Keywords:** acupuncture, network meta-analysis, stable chronic obstructive pulmonary disease, systematic review

## Abstract

**Background:**

Chronic obstructive pulmonary disease (COPD) has become a huge economic burden. Increasing randomized clinical trials have favored the efficacy of a specified kind of acupuncture-related therapies (ATs) for treating stable COPD. Nonetheless, comparative efficacy of different ATs for stable COPD remains unknown. Thus, the purpose of this network meta-analysis protocol aims to determine the optimal modality of ATs for treating stable COPD.

**Methods:**

Six Chinese and English mainstream databases, including PubMed, Cochrane Library, Embase, China Biomedical Literature Database, Chinese National Knowledge Infrastructure, and Wanfang Database, will be systematically retrieved. The time range of the literature search is from the establishment date of each database to July, 2021. The primary outcome measures will be pulmonary function and incidence of acute exacerbations. The secondary outcome measures will consist of 6-minute walking distance, St George's Respiratory Questionnaire, and COPD assessment test. The methodological quality of included studies will be evaluated by Cochrane risk-of-bias tool and the quality of evidence will evaluated through Grading of Recommendations Assessment, Development, and Evaluation instrument. Network meta-analysis will be completed using STATA software.

**Results:**

A synthesis of current evidence of ATs for stable COPD will be provided in this study.

**Conclusion:**

This study will contribute to synthesizing the evidence regarding the comparative efficacy of different modalities of ATs for stable COPD. Therefore, it will yield decision-making reference to further assist clinicians in determining the optimal modality of ATs in the treatment of stable COPD.

**Study Registration:**

This protocol was registered in the international prospective register of systematic reviews (PROSPERO) with the registration number CRD42020166649.

## 1 Introduction

Chronic obstructive pulmonary disease (COPD) is an incompletely reversible lung problem, which is characterized by persistent airway inflammation and airflow limitation.^[1]^ With a worldwide prevalence of 10.1%, COPD has become the third leading cause of death in the world, and its high prevalence, morbidity, and mortality lead to heavy burden for health-care systems.^[2,3]^ In the stable phase of COPD, patients presents symptoms as cough, exertional dyspnea, sputum production, chest tightness, or fatigue, which can significantly affect the exercise capacity and quality of life of patients.^[4]^ Pharmacotherapies and pulmonary rehabilitation (PR) are the conventional therapies for stable COPD, which are recommended by Global Strategy for the Diagnosis, Management, and Prevention of Chronic Obstructive Lung Disease.^[1,5]^ However, although symptoms can be managed to some extent for most patients by medications, the effects on the lung function decline and mortality in long term studies have been unsatisfactory.^[6]^ Besides, the side effects of pharmacotherapies cannot be underestimated, such as hoarseness, oropharyngeal candidiasis, and pneumonia.^[7,8]^ As for PR, it is reported only 1% to 2% of COPD patients can receive it as a routine treatment.^[9]^ And among the patients who have received PR, low adherence is another urgent problem remained to be solved. There are some special organizations that can provide help to supervise, but it costs higher medical expenses.^[10,11]^ Therefore, seeking other effective treatments for stable COPD to improve the efficacy and patient adherence as well as cut down the cost remains a challenging clinical problem.

Acupuncture-related therapies (ATs), as major parts of traditional Chinese medicine, have been indicated to be effective and safe.^[12,13]^ Acupuncture plays its role by stimulating the acupoints or specific points on body surface. There are different acupuncture modalities to stimulate acupoints, such as electricity (electroacupuncture), heat stimulation (moxibustion), and digital pressure (acupressure).^[14]^ Multiple clinical trials have indicated the effectiveness of a specified kind of ATs in the treatment of stable COPD.^[15–17]^

In recent years, the treatment regimen of ATs for stable COPD has been increasingly diversified, but evidence strength of relevant studies is somewhat inadequate in guiding the best treatment of stable COPD, bringing difficulties to clinicians to choose the best modality of ATs for treating stable COPD. At present, the majority of existing studies only evaluate evidence obtained by comparing a specific kind of ATs with other recognized active therapies for stable COPD, which is based on the method of conventional meta-analysis. Nonetheless, to date, there are no previously published studies that attempt to compare the efficacy of all common acupuncture modalities in clinical practice, which can be accomplished based on the method of a network meta-analysis (NMA). Moreover, Cochrane guidelines highly recommended that authors publish their protocols of meta-analysis or systematic review prior to performing a complete meta-analysis, which will significantly contribute to standardize procedures of meta-analysis and ensure the quality of the subsequent meta-analysis results in advance.

Taken together, this meta-analysis protocol will guide the upcoming NMA to compare the efficacy of multiple modalities of ATs to determine the optimal modality of ATs for treating stable COPD, so as to influence evidence-based treatment decisions for clinicians in the management of stable COPD.

## 2 Methods and analysis

### 2.1 Protocol registration and report

This protocol of NMA is designed and reported according to the Preferred Reporting Items for Systematic Reviews and Meta-analyses Protocols (PRISMA-P), which is uploaded in Supplementary File 1, Supplemental Digital Content. We have registered this protocol in the international prospective register of systematic reviews (PROSPERO) with the registration number CRD42020166649.

### 2.2 Eligibility criteria for included studies

#### 2.2.1 Types of studies

This review will be confined to all randomized controlled trials (RCTs) involving ATs for treating stable COPD.

#### 2.2.2 Eligibility of participants

Eligibility of participants will be defined as patients with stable COPD diagnosed by acknowledged criteria. We will apply no restrictions in terms of gender, age, region, or other demographic characteristics.

#### 2.2.3 Modalities of interventions

##### 2.2.3.1 Experimental interventions

ATs are defined as any acupuncture modalities that are commonly used in clinical practice, including manual acupuncture, electroacupuncture, acupoint injection, acupoint application, acupoint catgut embedding, warm needling, moxibustion, and other types, regardless of acupuncture materials, acupuncture techniques, and stimulation methods.

##### 2.2.3.2 Control interventions

Control interventions will include standard treatments for stable COPD (e.g., bronchodilator, expectorant, oxygen therapy, etc), no treatment or waiting-list, and sham controls (e.g., sham acupuncture).

#### 2.2.4 Types of outcome measurements

##### 2.2.4.1 Primary outcomes

(1) Pulmonary function: forced expiratory volume in 1 second/prediction (FEV_1_% pre) and forced expiratory volume in 1 second/forced vital capacity (FEV_1_/FVC%).
(2) Incidence of acute exacerbations.

##### 2.2.4.2 Secondary outcomes

(1) The 6-minute walking distance.
(2) St George's Respiratory Questionnaire.
(3) The COPD Assessment Test.

### 2.3 Literature search

We will search the following typical Chinese and English databases, including PubMed, Cochrane Library, Embase, China Biomedical Literature Database, Chinese National Knowledge Infrastructure, and Wanfang Database. The time range of the literature search is from the establishment date of each database to December, 2021. Language restrictions will not imposed. A combination of Medical Subject Heading and free-text terms incorporating database-specific controlled vocabularies and text words will be applied to search eligible studies in these electronic databases. In addition, the previous relevant reviews regarding ATs for stable COPD and reference lists of included studies will also be searched to identify additional studies. The preliminary search strategy is shown in Table 1, which will be modified based on specific syntax related requirements of each electronic database.

**Table 1:** Search strategy.

No.	Entry terms
1	exp “clinical trial”
2	(randomised or randomised). ab,ti.
3	placebo.ab,ti.
4	drug therapy.fs.
5	randomly.ab,ti.
6	trial.ab,ti.
7	groups.ab,ti.
8	or/1–7
9	animals
10	humans
11	9 not (9 and 10)
12	8 not 11
13	Lung Diseases, Obstructive
14	exp Pulmonary Disease, Chronic Obstructive
15	emphysema$.mp.
16	(chronic$ adj3 bronchiti$).mp.
17	(obstruct$ adj3 (pulmonary or lung$ or airway$ or airflflow$ or bronch$ or respirat$)).mp.
18	COPD.mp.
19	COAD.mp.
20	COBD.mp.
21	AECB.mp.
22	or/13-21
23	Acupuncture^∗^ or moxibustion^∗^ or scraping^∗^ or cupping^∗^ or warm needling^∗^
24	acupoint injection or acupoint application or acupoint catgut embedding
25	electroacupuncture^∗^ or electro-acupuncture^∗^
26	electro^∗^ NEXT acupuncture^∗^
27	electro^∗^ NEXT stimulation^∗^
28	acupressure^∗^
29	“transcutaneous electrical nerve stimulation”
30	acu^∗^ NEAR TENS
31	acu^∗^ NEAR transcutaneous^∗^
32	or/23-31
34	12 and 22 and 32

### 2.4 Study selection and data extraction

#### 2.4.1 Study selection

**Figure 1:**
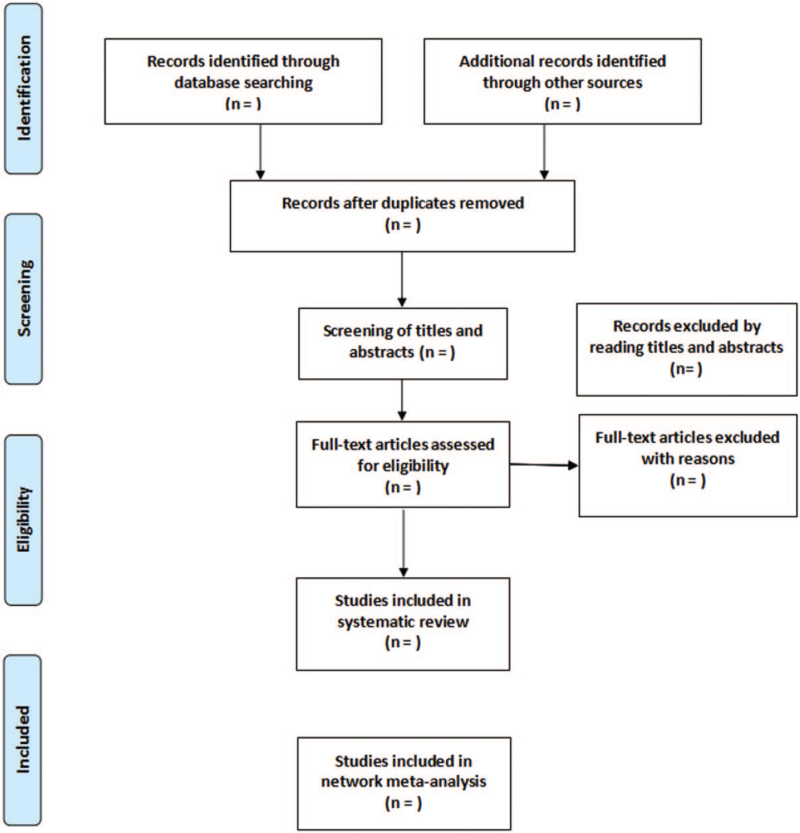
Flow diagram of study selection process.

We will use the EndNote X9 software (Thomson Reuters, Philadelphia, USA) to import the literature retrieved from the 6 databases. First, we will used the software to exclude duplicate articles, then 2 reviewers will independently browse the title and abstract, and exclude the articles that do not meet the requirements. If they cannot judge whether it is a qualified study, they need to read the full text to decide. After that, the 2 reviewers will cross-check whether the final selected research is consistent. If there is any difference between 2 reviewers during the selection of eligible studies, it will be decided through a group discussion with a third reviewer. The research selection process is shown in Figure 1.

#### 2.4.2 Data extraction and management

Two independent reviewers will extract the data from eligible studies using a standardized data extraction form. Data items from each included study will be extracted as follows: study characteristics: study title publication year, first author, journal, country, method of randomization method, blinding method, participant characteristics, interventions of the treatment and control group, primary and secondary outcome measures, etc. If there is any inconsistency between 2 reviewers during the data extraction, it will be verified by a third reviewer.

### 2.5 Quality assessment

The methodological quality for each included studies will be evaluated using the Cochrane risk-of-bias-tool^[18]^ by 2 reviewers independently. Any disagreements will be resolved by judgement by the third reviewer.

### 2.6 Statistical methods

#### 2.6.1 Management of missing data

If there is missing or incomplete data of any included study, the authors of the original study will be contacted by emails. If the authors do not respond to our request all the time, an intention-to-treat analysis will be employed to deal with missing data.

#### 2.6.2 Data synthesis and statistical methods

Before data are synthesized, the level of heterogeneity between included studies will measured by Cochrane Q statistic and the value of I^2^. If I^2^ ≤ 50% and *P* ≥ .05, it will suggest that heterogeneity is not important, in which case the fixed-effect model will be selected for meta-analysis. If I^2^ > 50% and *P* < .05, it will mean that the heterogeneity is significant, where the random-effect model will be selected for meta-analysis. Mean difference with 95% confidence intervals will be calculated for continuous variable data (e.g., FEV1%, FEV1/FVC%, acute exacerbation, 6-minute walking distance, St George's Respiratory Questionnaire, and COPD Assessment Test). Odds ratio or relative risk and associated 95% confidence intervals will be analyzed for categorical variable data.

NMA will be performed using the network command in Stata software (Stata V.14.0, StataCorp) to rank probabilities of different interventions. A network diagram will be made to visualize the numbers and interrelations of different interventions of ATs. The AT interventions results will be ranked according to their corresponding surface under the cumulative ranking curve, by evaluating the certainty extent of intervention superiority without resampling. The evaluation of the inconsistency between the direct and indirect comparison results will be based on the Z-test, the results of which will be displayed via a network graph. In addition, subgroup analyses will be conducted if significant heterogeneity is detected. Sensitivity analysis will be implemented by excluding studies of high risk of bias. Lastly, publication bias will be determined based on the funnel plots and Egger regression tests if sufficient numbers of RCTs are included.

#### 2.6.3 Grading the quality of evidence

According to the standard of the Grading of Recommendations Assessment, Development, and Evaluation system, the quality of evidence will be evaluated by 2 reviewers and it will be classified into 4 grades: “high”, “moderate”, “low”, and “very low”.^[19]^ If there was any disagreement, the final proposal will be selected through group discussion with the third reviewer.

## 3 Discussion

ATs are increasingly accepted by many countries. Being low-cost and generally side-effect-free, ATs are suitable for chronic diseases that need long-term treatment. The efficacy of ATs on stable COPD is supported by a number of relevant studies, but the modality of ATs used by different acupuncturists varies significantly, and their corresponding therapeutic effect is also uneven. Thus, it brings great difficulties to acupuncturists and patients for determining the optimal modality of ATs for treating stable COPD. In recent years, there have been more and more RCTs involving ATs for stable COPD, but there is a great lack of comparative study that aims to compare the efficacy of different modalities of ATs. Given that it will cost a lot of manpower and material resources to perform a RCT, based on existing eligible RCTs, we will use the method of NMA to summarize the direct and indirect evidence aiming to provide a ranking of multiple modalities of ATs for treating stable COPD.

Notably, it is highly recommended that authors publish their protocols of meta-analysis or systematic review prior to performing a complete meta-analysis based on the Cochrane guideline.^[20]^ In addition, we have registered this protocol of NMA in the online platform PROSPERO to avoid performance bias in the future. We will also conduct a rigorous selection and assessment of the outcomes to enhance the evidence for the results.

## 4 Conclusions

In summary, by comparing the efficacy of different modalities of ATs, this study is expected to determine the optimal modality of acupuncture therapy for treating stable COPD, thereby assisting patients and clinicians to choose the most appropriate acupuncture method in the treatment of stable COPD. It will also contribute to making recommendations as to evidence gaps, need for research, and good clinical practice points regarding acupuncture therapy for stable COPD.

## Author contributions

HJZ designed the trial protocol. ZTL drafted the manuscript. CW revised the manuscript. All the authors have read, revised and approved this version of the manuscript.

**Conceptualization:** Haijuan Zhang.

**Writing – original draft:** Zengtu Li.

**Writing – review & editing:** Jiali Lou, Chao Wang.

## Supplementary Material

Supplemental Digital Content
